# Deep Brain Stimulation Electrode Reconstruction: Comparison between Lead-DBS and Surgical Planning System

**DOI:** 10.3390/jcm12051781

**Published:** 2023-02-23

**Authors:** Yichen Xu, Guofan Qin, Bojing Tan, Shiying Fan, Qi An, Yuan Gao, Houyou Fan, Hutao Xie, Delong Wu, Huanguang Liu, Guang Yang, Huaying Fang, Zunyu Xiao, Jianguo Zhang, Hua Zhang, Lin Shi, Anchao Yang

**Affiliations:** 1 Department of Functional Neurosurgery, Beijing Tiantan Hospital, Capital Medical University, Beijing 100070, China; 2 Department of Neurosurgery, Capital Institute of Pediatrics, Beijing 100020, China; 3 Department of Neurosurgery, The First Affiliated Hospital of Harbin Medical University, Harbin 150007, China; 4 Beijing Advanced Innovation Center for Imaging Theory and Technology, Capital Normal University, Beijing 100089, China; 5 Academy for Multidisciplinary Studies, Capital Normal University, Beijing 100089, China; 6 Molecular Imaging Research Center, Harbin Medical University, Harbin 150076, China; 7 Beijing Neurosurgical Institute, Capital Medical University, Beijing 100070, China

**Keywords:** deep brain stimulation, Lead-DBS, surgery planning system, electrode localization, contact coordinate, DBS programming

## Abstract

Background: Electrode reconstruction for postoperative deep brain simulation (DBS) can be achieved manually using a surgical planning system such as Surgiplan, or in a semi-automated manner using software such as the Lead-DBS toolbox. However, the accuracy of Lead-DBS has not been thoroughly addressed. Methods: In our study, we compared the DBS reconstruction results of Lead-DBS and Surgiplan. We included 26 patients (21 with Parkinson’s disease and 5 with dystonia) who underwent subthalamic nucleus (STN)-DBS, and reconstructed the DBS electrodes using the Lead-DBS toolbox and Surgiplan. The electrode contact coordinates were compared between Lead-DBS and Surgiplan with postoperative CT and MRI. The relative positions of the electrode and STN were also compared between the methods. Finally, the optimal contact during follow-up was mapped onto the Lead-DBS reconstruction results to check for overlap between the contacts and the STN. Results: We found significant differences in all axes between Lead-DBS and Surgiplan with postoperative CT, with the mean variance for the X, Y, and Z coordinates being −0.13, −1.16, and 0.59 mm, respectively. Y and Z coordinates showed significant differences between Lead-DBS and Surgiplan with either postoperative CT or MRI. However, no significant difference in the relative distance of the electrode and the STN was found between the methods. All optimal contacts were located in the STN, with 70% of them located within the dorsolateral region of the STN in the Lead-DBS results. Conclusions: Although significant differences in electrode coordinates existed between Lead-DBS and Surgiplan, our results suggest that the coordinate difference was around 1 mm, and Lead-DBS can capture the relative distance between the electrode and the DBS target, suggesting it is reasonably accurate for postoperative DBS reconstruction.

## 1. Introduction

Deep brain stimulation (DBS) has become a standard surgical intervention for movement disorders, including Parkinson’s disease (PD) and dystonia (DT) [1]. The surgical procedure for DBS involves placing an electrode in the target nucleus of the brain and delivering sustained high-frequency stimulation to the target nucleus, thereby alleviating the symptoms. In terms of optimizing the therapeutic effects of DBS, accurate placement of the electrode is critical [2]. In patients with PD, a deviation from the preoperatively defined target, such as the subthalamic nucleus (STN), can significantly impair postoperative effects [3,4,5]. More importantly, the relative location of the DBS electrode with respect to the target nucleus is crucial to the surgery because it can guide the DBS programming, with this especially holding true for the future directional lead [6,7]. However, an accurate postoperative DBS electrode reconstruction method remains an intractable problem in the clinical practice of functional neurosurgery.

A conventional electrode localization method involves fusing the postoperative computerized tomography (CT) or magnetic resonance imaging (MRI) with the preoperative MRI. This can be achieved using a surgical planning system such as the Surgiplan system (ELEKTA Instruments AB). The coordinates of the electrode contacts can be extracted and compared with the pre-defined target to determine the accuracy of the electrode placement. This is a relatively accurate method because it is based on an individual image that can preserve individual variability, which has been widely used in clinical setting. Nevertheless, there are several inherent weaknesses to this method, including the intense labor burden and the low tissue contrast of postoperative CT. It might, therefore, be helpful to use postoperative MRI, although several studies suggested that artifacts in postoperative MRI may significantly distort the images [8,9]. Furthermore, even though studies have demonstrated the safety of postoperative MRI in patients undergoing DBS [10,11], postoperative MRI still carries risks in such patients. 

Several toolboxes for identifying and reconstructing the DBS electrode placement in a semi-automated manner have been developed [12]. Among these toolboxes, the MATLAB-based toolbox ‘Lead-DBS’ has become the most established tool for postoperative DBS reconstruction [13,14]. Lead-DBS reconstruction standardizes and normalizes the electrode using postoperative CT and preoperative MRI, with an algorithm automatically calculating the electrode coordinates. Lead-DBS can display the relative spatial position of the electrode and target nucleus, adding additional information to the electrode localization. The accuracy of Lead-DBS has been verified by several research teams, and Ewert et al. showed that the nonlinear registration method in Lead-DBS is comparable to manual segmentation [15]. Furthermore, the advanced normalization tools (ANTs) that is frequently used in the normalization step of Lead-DBS was also shown to be reliable [16]. These studies suggest that Lead-DBS gives reliable DBS reconstruction results. Moreover, Lofredi et al. demonstrated that the variance of Lead-DBS across different users was around 0.6 mm, reflecting the accuracy of the toolbox [17]. 

In this study, we further evaluated the accuracy of the Lead-DBS reconstruction method by comparing it with the DBS reconstruction method using a surgical planning system (Surgiplan). We assessed the accuracy of Lead-DBS by comparing DBS reconstruction results in the anterior commissure–posterior commissure (AC-PC) coordinate system between Lead-DBS and Surgiplan with different postoperative imaging modalities. We also measured the Euclidean distance between the electrode tip and the pre-defined surgical target point, and the relative distance between the electrode and the STN to determine the accuracy of electrode localization in the different methods. Finally, we mapped the optimal contacts during programming onto the Lead-DBS reconstruction results to assess the overlap between the optimal contacts and the target nucleus.

## 2. Materials and Methods

### 2.1. Patients and Preoperative Assessment

Twenty-one patients with PD and five patients with DT who received bilateral STN DBS surgery at Beijing Tiantan Hospital, Capital Medical University, between December 2021 and January 2022 were retrospectively enrolled in our study. All patients were in an advanced stage of disease and met the criteria for DBS implantation [18]. All patients provided written informed consent, and the study was approved by the Institutional Review Board of Beijing Tiantan Hospital (KY-2022-139-02). Two senior neurologists independently completed the preoperative assessments of all patients. For patients with PD, the Movement Disorder Society-Unified Parkinson’s Disease Rating Scale Part III (MDS-UPDRS-III, on/off medication) was used to assess their motor symptoms. For those with DT, the assessments included the Burke–Fahn–Marsden Dystonia Rating Scale (BFMDRS) and Global Rating Scale (GRS). None of the patients with DT took medication for the treatment of DT symptoms. 

### 2.2. Surgical Procedures

A routine 3.0-T high-resolution MRI acquisition with 3D T1-weighted and T2-weighted pulse sequences was scheduled for all patients prior to surgery. All patients were in the off-medication state for at least 12 h prior to surgery. On the morning of the surgery, a stereotactic Leksell frame (Elekta Instruments AB, Stockholm, Sweden) was mounted on the patient’s head under local anesthesia and full head CT (slice thickness 0.625 mm) was acquired. In the operating theater, the preoperative MRI and CT data were imported into the Surgiplan system and fused together. The target and trajectory were determined based on direct visualization of the STN on T2 and T1 sequences, respectively [19]. The target coordinates were based on the AC-PC system, with the PC selected as the origin for the coordinates. During surgery, the patients were fully awake with local anesthesia and sedation. Dural puncture was used in the dura opening. Our previous study shows that dural puncture can reduce pneumocephalus and brain shift [20]. Microelectrode recording (MER, NeuroOmega, Israel) was used to guide the advancement of the microelectrode during surgery. The microelectrode was considered to have entered the STN when the typical STN electrophysiological signal was present during MER. This signal was characterized by an increase in the background noise and an irregular discharge pattern with occasional bursts. A quadripolar electrode (Medtronic 3389 or PINS 301) was implanted bilaterally and intra-operative stimulation was delivered to evaluate symptom improvement and adverse events. For patients with PD, an implantable pulse generator (IPG) was implanted into the subclavian fossa after electrode implantation. In patients with DT, the electrode was externalized for 1 week to measure clinical improvement, and an IPG was implanted if satisfactory clinical improvement was achieved. 

### 2.3. Postoperative Electrode Localization

#### 2.3.1. Electrode Reconstruction in Surgiplan

**Figure 1:**
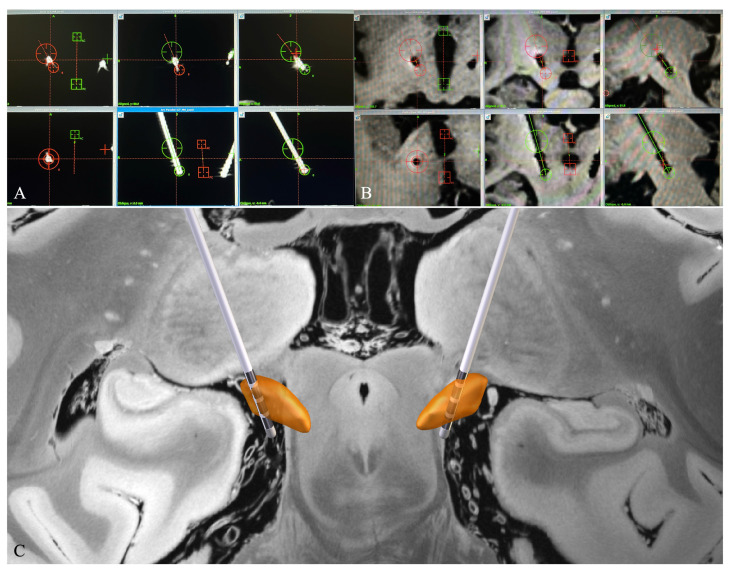
DBS electrode reconstruction using Surgiplan and Lead-DBS. Reconstruction of the DBS electrode in an example patient using Surgiplan with postoperative CT (**A**), Surgiplan with postoperative MRI (**B**), and Lead-DBS toolbox (**C**). In postoperative CT and MRI, the electrode is represented by a signal with a different intensity; in postoperative CT, the electrode is represented by a hyperintense electrode artifact (**A**), whereas in postoperative MRI, the electrode is estimated as an imaginary center of the hypointense signal (**B**).

A routine postoperative CT scan was scheduled for all patients after surgery to check the electrode localization and for the presence of intracranial hemorrhage. This postoperative CT was later imported into the Surgiplan system and rigidly fused with the preoperative MRI to obtain the stereotactic coordinates of the electrode contacts in the AC-PC system (Figure 1A). Two senior neurosurgeons conducted a thorough visual inspection of the fused image to determine the center point of the most ventrally distributed electrode contact, which was then marked, and its coordinates were obtained in the AC-PC system. Positive X, Y, and Z coordinates indicate lateral (right), anterior, and inferior directions towards the PC, respectively. Then, another point along the trajectory of the cranial part of the electrode without obvious bending was selected, and the line equation of the electrode was calculated from the coordinates of these two points as follows:

$$\frac{x-x1}{x-x2}=\frac{y-y1}{y-y2}=\frac{z-z1}{z-z2}$$

where A (x1, y1, z1) = the coordinates of the most ventral contact, and B (x2, y2, z2) = the coordinates of another point along the electrode trajectory.

The DBS electrode implanted in the patients was a Medtronic 3389 or PINS 301, and the distance between the two adjacent contact pairs was 2 mm (from the center of one contact to the corresponding point of the adjacent one). The second, third, and fourth electrode contacts were located along the trajectory at 2, 4, and 6 mm, respectively, away from the ventral-most electrode contact. The equation used to calculate the other three electrode contacts was as follows:

$$x_{i}=x1-\sqrt{l_{i}{}^{2}-{\left(y1-y_{i}\right)}^{2}-{\left(z1-z_{i}\right)}^{2},}$$

$$y_{i}=y1-\sqrt{l_{i}{}^{2}-{\left(x1-x_{i}\right)}^{2}-{\left(z1-z_{i}\right)}^{2},}$$

$$z_{i}=z1-\sqrt{l_{i}{}^{2}-{\left(x1-x_{i}\right)}^{2}-{\left(y1-y_{i}\right)}^{2}},$$

where p (*x*1, *y*1, *z*1) = the coordinates of the ventral-most contact, q ($x_{i}$, $y_{i}$, $z_{i}$) = the coordinates of the electrode contact i at distances of 2, 4, and 6 mm from the ventral-most electrode contact, respectively, and $l_{i}$ = the distance from the electrode contact i to the ventral-most electrode contact. 

To evaluate the influence of a different image dataset on electrode reconstruction, the DBS electrode was also reconstructed in Surgiplan using the postoperative MRI. The data from the postoperative MRI scheduled 2 days after the surgery were imported into the Surgiplan system to calculate the electrode contact coordinates in a similar way to that mentioned above. However, not all patients underwent postoperative MRI, with MRI data being available for only 11 of them. In MRI, the electrode is represented as a hypointense signal (Figure 1B). The same two senior neurosurgeons first located the ventral-most contact. A previous study suggests that the ventral-most contact is a suitable reference in calculating the electrode contact coordinate in postoperative MRI [21]. According to the manufacturer, the distance between the electrode tip and the ventral-most contact is 1.5 mm in both the Medtronic 3389 and PINS 301 DBS electrode. An additional 0.75 mm (half-length of the stimulating contact in Medtronic 3389 and PINS 301 electrode) was added to locate the center point of the ventral-most contact. Then, the coordinate of the ventral-most contact was calculated using the same equation listed above. Subsequently, the coordinate of the other contacts in postoperative MRI was deduced in the same way mentioned above.

Finally, the four contact coordinates of each hemisphere were averaged before further analysis. 

#### 2.3.2. Electrode Reconstruction in Lead-DBS

The preoperative MRI and postoperative CT data of each patient were imported into Lead-DBS and the electrode reconstruction procedure was performed in accordance with the methods of Horn et al. [12,13] (Figure 1C). We chose the default settings in Lead-DBS reconstruction because they give the most accurate results [17]. Briefly, postoperative CT scans were co-registered to the preoperative T1 scans, which were then normalized into the Montreal Neurological Institute (MNI) space using the Advanced Normalization Tools (ANTs) [22]. Following co-registration and normalization, the DBS electrode was extracted from the post-operative CT data and localized in template space using the refined TRAC/CORE method [13]. The electrode was projected into the template space against the DISTAL atlas [23]. The contact coordinates were then converted and averaged into the AC-PC system using the Lead-DBS plug-in [24].

### 2.4. Relative Position between the Electrode and the DBS Target

The relative position between the electrode and the DBS target was evaluated both qualitatively and quantitatively at the maximum level of the red nucleus (RN). One electrode from the right hemisphere and one electrode from the left hemisphere were excluded from this analysis because these electrodes were located above the maximum RN level. Two neurosurgeons independently reviewed the Lead-DBS reconstruction results and the Surgiplan fused postoperative CT and preoperative T2-weighted MRI image of each patient (Supplementary Figure S2). The consistency of the relative positions of the STN and the center point of the electrode between Lead-DBS and Surgiplan was manually evaluated by the two neurosurgeons and represented as the percentage number. Furthermore, the intersection point of the straight line connecting the center of the RN and the electrode with the ventral border of the STN was selected in Lead-DBS and Surgiplan. The relative distance between the electrode and STN was calculated as the Euclidean distance between the STN intersection point and the electrode. The ventral border of the STN was selected as the reference for calculating the relative distance between the electrode and the STN because it can be more clearly identified than the lateral border in MRI T2 sequences. The maximum RN level, the RN center point, and the intersection point were calculated using customized scripts in Lead-DBS with the DISTAL atlas, and were manually selected in Surgiplan by the same two neurosurgeons using the fused image.

### 2.5. Euclidean Distance between the Electrode Tip and the Pre-Defined Target

In our center, the final electrode placement is based on the preoperatively defined target and intraoperative MER guidance. All MER recordings were acquired using the NeuroOmega system (Alpha Omega Engineering, Nazareth Israel), and only one microelectrode was used during MER recording; thus, it shares the same trajectory as the DBS electrode. The MER distance recorded in the NeuroOmega system, which can be directly obtained in the NeuroOmega system, is the relative distance between the electrode tips and the preoperatively defined target. It would be inaccurate to directly compare the coordinates of the ventral-most contact with the coordinates of the preoperative target because the final electrode implantation site may vary depending on the MER signal. Therefore, the Euclidean distance between the electrode tip and the preoperatively defined target (serving as the reference point) was calculated, and the accuracy of the two electrode localization methods was determined by comparing with the intraoperative MER-recorded distance. Notably, the MER-recorded distance is the length between the electrode tip and the target. Thus, the distance between the ventral-most contact and electrode tip according to the manufacturer was added before the comparison. 

### 2.6. DBS Parameter Programming and Patient Follow-Up

All DBS parameter programming began at 1 month after surgery. Each DBS contact was activated using the following parameters: monopolar configuration, 1.2–1.6 V, 60 μs, and 130–140 Hz. The motor symptoms of all patients were evaluated by the same movement disorder neurologist using MDS-UPDRS-III for patients with PD and BFMDRS/GRS for patients with DT. In the patients with PD, the motor symptoms were evaluated in both the off- and on-medication conditions, and the optimal contact was defined as the active contact that yielded the highest motor improvement according to MDS-UPDRS-III and was without side effects. In the patients with DT, the optimal contact definition was made in a rather subjective manner based on the patient’s feedback because the improvement in DT syndromes is not immediate. The optimal contacts were then mapped onto the DBS reconstruction results, along with the STN based on the DISTAL atlas [23]. The overlaps of the optimal contacts and the STN and its subregions were assessed by two neurosurgeons with manual visualization. 

### 2.7. Data Analysis and Statistics

The electrode trajectory, coordinates, and relative distance were calculated using customized scripts written in MATLAB 2020b (Mathworks, Natick, MA, USA). All continuous data are presented as mean ± standard deviation (SD). Data in tables are expressed in millimeters. The coordinates of the four contacts were averaged in each hemisphere of each patient before comparison. The normal distribution of the data was assessed with the Kolmogorov–Smirnov test. The coordinates obtained using Lead-DBS and Surgiplan with postoperative CT data were compared using a paired *t*-test or Wilcoxon paired signed rank test. A paired Tukey’s multiple comparison test or Dunn’s multiple comparison test (for non-normally distributed data) was used to compare the Euclidean distance and coordinates between Lead-DBS, Surgiplan with postoperative CT, and Surgiplan with postoperative MRI. The relative distance between the electrode and the DBS target was compared between Lead-DBS and Surgiplan using a paired *t*-test. The preoperative and postoperative MDS-UPDRS-III and BFMDRS scores were compared using two sample *t*-tests. The level of significance was set at *p* < 0.05. The statistical analysis was performed using SPSS (version 20.0; IBM Corp., Armonk, NY, USA).

## 3. Results

### 3.1. Patient Characteristics

Among the 26 included patients, 21 were diagnosed with PD and 5 were diagnosed with DT. The mean patient age and mean age of disease onset were 58.4 ± 7.4 years and 51.1 ± 7.4 years, respectively. In the preoperative assessment, the mean off-medication MDS-UPDRS part-III score was 44.7 ± 18.9 and the improvement rate after levodopa intake was 55.4 ± 16.7%. Among the patients with DT, four were diagnosed with Meige syndrome and one patient was diagnosed with cervical DT. The mean BFMDRS sum score and GRS in the DT group were 22.7 ± 15.1 and 23 ± 6.7, respectively. The patient characteristics are summarized in Table 1. 

### 3.2. Comparison of the Coordinates between Lead-DBS and Surgiplan with Postoperative CT

**Figure 2:**
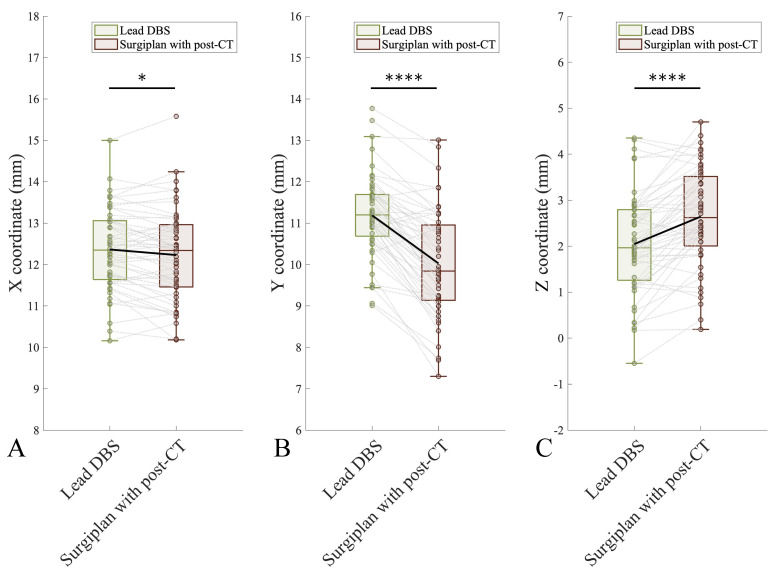
Comparison of electrode contact coordinates between Lead-DBS and Surgiplan with postoperative CT. A significant difference was found in the X (**A**), Y (**B**), and Z (**C**) coordinates between Lead-DBS and Surgiplan (X coordinate, *p* = 0.0251; Y coordinate, *p* < 0.0001; Z coordinate, *p* < 0.0001; all paired *t*-test). *: *p* < 0.05, ****: *p* < 0.0001.

We first pooled all the X, Y, and Z coordinates of the electrode contacts together (using the absolute values of the X coordinates on different sides) and compared the difference between Lead-DBS and Surgiplan with postoperative CT. We found significant differences in the X, Y, and Z coordinates between the methods (Figure 2; X coordinate, *p* = 0.0251; Y coordinate, *p* < 0.0001; Z coordinate, *p* < 0.0001; all paired *t*-tests). The mean discrepancies between the coordinates obtained using Lead-DBS and Surgiplan with postoperative CT were −0.13, −1.16, and 0.59 mm in the X, Y, and Z coordinates, respectively (Surgiplan minus Lead-DBS).

**Figure 3:**
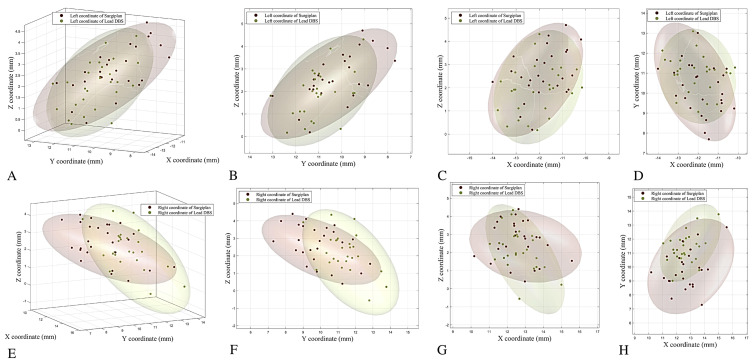
Coordinate distribution in the AC-PC system. The distributions of the coordinates from each patient with Lead-DBS and Surgiplan with postoperative CT are shown in the AC-PC system. The distributions of the left (**A**) and right (**E**) electrode contact coordinates are shown in 3D space. The left electrode coordinate distribution is also shown in left to right (**B**), posterior to anterior (**C**), and inferior to superior (**D**) directions. Similarly, the right electrode coordinate distribution is also shown in right to left (**F**), posterior to anterior (**G**), and inferior to superior (**H**) directions.

We further compared the coordinates of the electrode contacts separately for the left and right hemispheres. In the right hemisphere, the Y and Z coordinates obtained using Lead-DBS and Surgiplan with postoperative CT were significantly different, but the X coordinates were not. In the left hemisphere, the X, Y, and Z coordinates calculated using Lead-DBS and Surgiplan with postoperative CT were all significantly different (Supplementary Figure S1; Right X coordinate, *p* = 0.8019; Right Y coordinate, *p* < 0.0001; Right Z coordinate, *p* = 0.0012; Left X coordinate, *p* < 0.0001; Left Y coordinate, *p* < 0.0001; Left Z coordinate, *p* < 0.0001; all paired *t*-test). Furthermore, we found that the distribution of the coordinates was less inferior and more lateral and anterior with Lead-DBS than with Surgiplan (Figure 3). The detailed coordinate information is listed in Table 2. 

### 3.3. Comparison of Calculated Coordinates between Lead-DBS, Surgiplan with Postoperative CT, and Surgiplan with MRI

Postoperative MRI was available for 11 patients in our dataset. We found that the Y and Z coordinates showed significant differences between Lead-DBS and Surgiplan with postoperative CT or MRI (Supplementary Figure S2B,C; Y coordinate: Lead-DBS vs. Surgiplan with postoperative CT, *p* < 0.0001; Lead-DBS vs. Surgiplan with postoperative MRI, *p* < 0.0001; Surgiplan with postoperative CT vs. Surgiplan with postoperative MRI, *p* = 0.9994. Z coordinate: Lead-DBS vs. Surgiplan with postoperative CT, *p* < 0.0001; Lead-DBS vs. Surgiplan with postoperative MRI, *p* < 0.0001; Surgiplan with postoperative CT vs. Surgiplan with postoperative MRI, *p* = 0.4171; all paired Tukey’s multiple comparisons tests). For the X coordinates, the differences between Lead-DBS and Surgiplan with postoperative CT or MRI did not reach statistical significance, despite a significant difference being found between Surgiplan with postoperative CT and Surgiplan with postoperative MRI (Supplementary Figure S2A; X coordinate: Lead-DBS vs. Surgiplan with postoperative CT, *p* = 0.3367; Lead-DBS vs. Surgiplan with postoperative MRI, *p* = 0.4322; Surgiplan with postoperative CT vs. Surgiplan with postoperative MRI, *p* = 0.038; all paired Tukey’s multiple comparisons tests). The detailed coordinate information is listed in Supplementary Table S1.

### 3.4. Relative Position of the Electrode and the DBS Target

**Figure 4:**
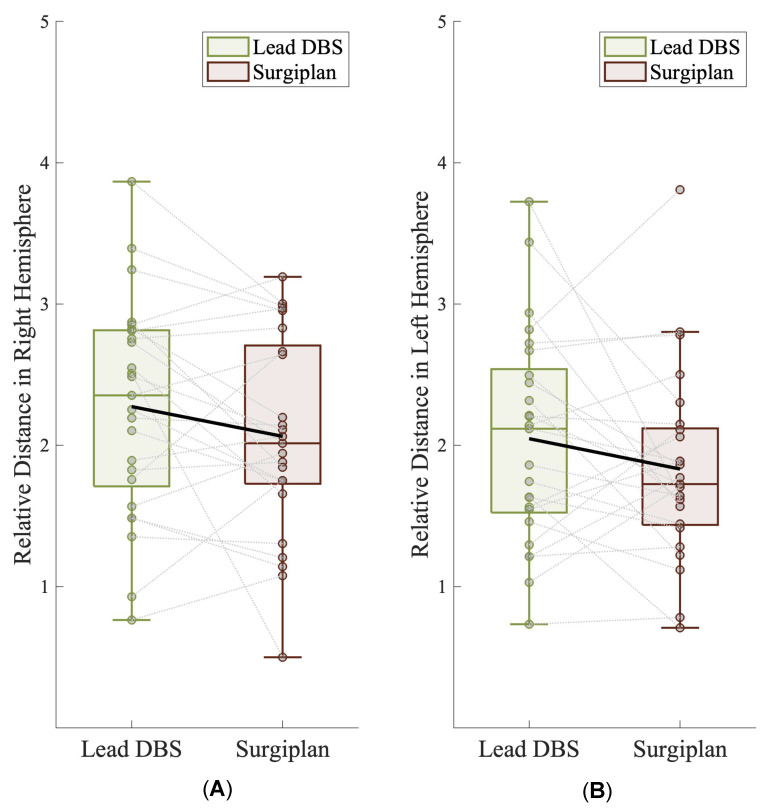
Relative distance between the electrode and the STN in Lead-DBS and Surgiplan. The relative distance between the electrode and the STN in Lead-DBS and Surgiplan did not differ significantly in right (**A**) or left (**B**) hemispheres (right hemisphere, paired *t*-test, Lead-DBS relative distance vs. Surgiplan relative distance, 2.27 ± 0.75 vs. 2.06 ± 0.71, *p* = 0.105; left hemisphere, paired *t*-test, Lead-DBS relative distance vs. Surgiplan relative distance, 2.05 ± 0.76 vs. 1.83 ± 0.67, *p* = 0.159).

Subsequently, we asked the question of whether the relative position between the electrode and the STN was consistent between the Lead-DBS reconstruction and Surgiplan with the fused image in each patient. We found that 72.5% (29/40) of the Lead-DBS reconstructions were consistent with Surgiplan (Supplementary Figure S3). When Surgiplan was used with the fused postoperative image, we found that two electrodes (one from the right hemisphere and one from the left hemisphere) were localized above the maximum RN level. These two electrodes were also located above the maximum RN level in the Lead-DBS reconstruction, thereby indicating the accuracy of Lead-DBS. We further calculated the relative distance between the electrodes and the ventral border of the STN in Lead-DBS and Surgiplan (see Methods), and found that the relative distance between the electrodes and the STN did not differ significantly between Lead-DBS and Surgiplan (Figure 4A,B; right hemisphere, Lead-DBS relative distance vs. Surgiplan relative distance, 2.27 ± 0.75 vs. 2.06 ± 0.71, *p* = 0.105; left hemisphere, Lead-DBS relative distance vs. Surgiplan relative distance, 2.05 ± 0.76 vs. 1.83 ± 0.67, *p* = 0.159; all paired *t*-test), suggesting that Lead-DBS was able to capture the relative position between the electrode and the DBS target.

### 3.5. Validation of Lead-DBS According to the Euclidean Distance and Clinical Follow-Up Data

To validate the accuracy of the electrode localization method, we compared the MER-recorded distance with the Euclidean distance between the preoperative defined target and the electrode tip in Lead-DBS and Surgiplan with postoperative CT. A paired multiple comparisons test indicated that the Euclidean distances calculated using Lead-DBS and Surgiplan with postoperative CT did not differ significantly from the MER-recorded distances (Supplementary Figure S4; Lead-DBS distance vs. Surgiplan distance, *p* > 0.9; Lead-DBS distance vs. MER distance, *p* > 0.9; Surgiplan distance vs. MER distance, *p* > 0.9; all paired Dunn’s multiple comparison test). The detailed coordinate information is listed in Supplementary Table S2.

To further validate the accuracy of the Lead-DBS electrode localization, we mapped the optimal contact onto the Lead-DBS reconstruction results using the STN atlas. Manual identification suggested that all optimal contacts (according to the follow-up data) overlapped with the STN in the Lead-DBS reconstruction results. Additionally, we found that 73.8% (31/42) of the optimal contacts were located in the dorsolateral region of the STN in patients with PD, and 70% (7/10) were located in the dorsolateral region of the STN in patients with DT (Supplementary Figure S5A,B). In PD, the optimal contact stimulation significantly alleviated the motor symptoms compared with the preoperative baseline, both in the off- and on-medication states (Supplementary Figure S5C; preoperative off-medication MDS-UPDRS-III vs. postoperative off-medication MDS-UPDRS-III: 43.7 ± 19.3 vs. 21.6 ± 11.4, *p* = 0.002; preoperative on-medication MDS-UPDRS-III vs. postoperative on-medication MDS-UPDRS-III: 21.2 ± 12.5 vs. 10.9 ± 5.6, *p* = 0.0135). Nevertheless, the motor symptom improvements were similar between patients with both active contacts in the dorsolateral region of STN and patients with at least one active contact outside the dorsolateral region of the STN (Supplementary Figure S6; postoperative off-medication MDS-UPDRS-III: 21.8 ± 10.7 vs. 21.3 ± 11.1, *p* = 0.98; postoperative on-medication MDS-UPDRS-III: 10.4 ± 4.63 vs. 10.5 ± 5.2, *p* = 0.77). In the patients with DT, we found a decrease in the BFMDRS and GRS scores after stimulation with the optimal contact, although the differences did not reach statistical significance (Supplementary Figure S5D; preoperative BMFDRS vs. postoperative BMFDRS: 22.5 ± 15.2 vs. 10.4 ± 4.4, *p* = 0.116; preoperative GRS vs. postoperative GRS: 23 ± 6.7 vs. 14 ± 7.6, *p* = 0.08). 

## 4. Discussion

DBS surgery has become a standard treatment for drug-resistant movement disorders [25]. One critical factor determining the success of the surgery is accurate electrode placement, and a reliable postoperative DBS localization method is required. Lead-DBS has been continuously optimized and has become one of the most-used DBS reconstruction methods. It can visualize the electrode along with the target nucleus, save on manpower requirements, and allow data analysis across different patients. Several studies have verified the accuracy of each step in the Lead-DBS reconstruction, and it remains reliable regardless of the experience level of users [15,16,17]. On the basis of these findings, we further validated the accuracy of Lead-DBS by comparing it with a DBS reconstruction method using the Surgiplan surgical planning system. To our knowledge, the postoperative DBS reconstruction of Lead-DBS has not been previously validated in comparison with a surgical planning system, and our study provides additional value regarding the accuracy of Lead-DBS. 

In this study, we found significant differences in the X, Y, and Z coordinates of DBS reconstructions between Lead-DBS and Surgiplan. There are several potential explanations for these discrepancies between the two DBS reconstruction methods. Although both methods may be influenced by co-registration error, an additional error is introduced into Lead-DBS by the non-linear ANTs registration. Meanwhile, variance in the normalization should also be taken into consideration, even though the default settings of Lead-DBS yield comparable results to expert segmentation [15]. Furthermore, the coordinate calculations in Lead-DBS are achieved by an algorithm that models the lead trajectory as a straight line and places the electrode contacts at equal distances along the line [13]. However, the electrodes may not actually be placed in an ideal straight line because they may bend after implantation [26,27]. To obtain maximum accuracy, we calculated the contact coordinates using the line equation for the best fit for the cranial part of the electrode. The cranial part of the electrode, which contains four contacts, is less likely to bend after surgery, thereby minimizing the influence of electrode bending. The conversion of the coordinate system is another source of possible errors [24], as is the possibility of brain shift, which is mainly caused by cerebral spinal fluid (CSF) leakage and intracranial air [28]. Nathanael et al. demonstrated that air volumes can lead to deviation in the electrode positioning and recommended performing DBS programming 4 weeks after surgery [29]. In Surgiplan, the influence of brain shift cannot be avoided if the postoperative images are scheduled a few days after surgery. In comparison, Lead-DBS is able to correct brain shift and may be of benefit in patients with a high degree of brain shift [12]. In our study, intracranial air did not influence the results to a great extent because we performed the dura puncture using monopolar coagulation instead of a dura cruciate incision. Our previous study demonstrated that this technique significantly reduces air volumes and brain shift [20]. Meanwhile, we focused more on the accuracy of the early electrode identification. This early DBS reconstruction result is equally crucial in that it allows the neurosurgeon to make timely clinical decisions such as reoperation if the electrode is implanted unsatisfactorily. It also provides accurate electrode location information in patients requiring externalization and ward testing. Nevertheless, despite the significant variance in contact coordinates, the averaged error between Lead-DBS and Surgiplan was only around 1 mm. Studies have shown that an error of 2–3 mm in DBS is acceptable in clinical practice; thus, the error we found in the Lead-DBS can be considered reasonably low and may not significantly influence the accuracy of Lead-DBS [30,31].

We also investigated whether different post-operative modalities influence DBS reconstruction. Compared with MRI, CT is more sensitive and is much better for visualizing the electrode, despite its low tissue contrast [9]. In comparison, postoperative MRI is able to show the electrode’s trajectory while maintaining high tissue resolution [8]. In our study, we found a significant difference in Y and Z coordinates between Lead-DBS and Surgiplan with both modalities, and higher variance in Z coordinates was found between Lead-DBS and Surgiplan with postoperative MRI. Similarly, Lofredi et al. also found high variance in electrode identification using a postoperative MRI dataset [17]. This high variance in electrode localization using postoperative MRI may be blamed on the nature of the currently used MRI sequence. It was found that electrode artifacts in MRI were larger than CT, and that tissue distortion makes electrode contact identification less reliable [8,32]. Furthermore, magnetic susceptibility effects may induce more artifacts in a T2 sequence, and not all DBS models support MRI scans [10,33,34,35]. Nevertheless, with the future development of MRI sequences and DBS electrode materials, postoperative MRI is likely to become a more reliable method for locating DBS electrodes. Subsequently, we found that the averaged variance between Surgiplan with postoperative CT and MRI only existed in the X coordinates and was less than 1 mm, which is similar to previous studies [8,36,37]. The significant difference in the X coordinates may indicate the difference in how electrodes are perceived in the MRI and CT. However, Lee et al. demonstrated a significant difference in electrode localization between postoperative MRI and CT [30]. This inconsistency may be explained by our use of 3.0-T MRI, rather than the 1.5-T MRI that Lee et al. used, which may result in lower image contrast. As postoperative CT and MRI are almost equally accurate for electrode contact localization, postoperative CT should currently be recommended because of the superiority for electrode visualization [38]. Additionally, we found that the fused postoperative CT and preoperative T2-weighted MRI images could clearly demonstrate both the electrode and STN (Supplementary Figure S3). It may be optimal to use the fused postoperative CT and preoperative T2-weighted MRI images to evaluate and display the relative position of the electrode with the visible DBS target such as STN.

Although the contact coordinates differed significantly between Surgiplan and Lead-DBS, the differences only had a minor influence on the localization of the electrode. Our results showed that the Euclidean distance between the electrode tip and the preoperatively defined target points calculated using Lead-DBS and Surgiplan were not significantly different from the MER-recorded distance, suggesting the accuracy of electrode localization. At the patient level, we also found that the relative position between the electrode and the STN in most of the Lead-DBS reconstructions was consistent with that of Surgiplan using the postoperative fused image. In addition, the relative distance between the electrode and the STN did not differ significantly between Lead-DBS and Surgiplan at the group level. These results suggest that Lead-DBS was able to preserve the relative position between the electrode and the DBS target. We also mapped the optimal contacts onto the Lead-DBS reconstruction results during patient follow-up and found that all optimal contacts were located within the target nucleus, and that the majority of the optimal contacts overlapped with the dorsolateral region of the STN. The dorsolateral region of the STN is considered to be the sensorimotor subregion, and evidence suggests that stimulation of this sensorimotor region can significantly alleviate motor symptoms [39,40]. Our mapping results suggest that Lead-DBS may have additional value in DBS programming and may be treated as a supplement in DBS programming. Currently, a data-driven algorithm for DBS programming based on Lead-DBS reconstruction has been developed, and a crossover trial suggested that this automated DBS programming offers comparable performance to manual programming but with less time cost [41,42]. Our findings concerning the electrode–target position and patient follow-up indicated that Lead-DBS is reasonably accurate for determining the relative position of the electrode and the target, adding more credibility to the Lead-DBS-based programming algorithm. However, the use of Lead-DBS for parameter programming still carries concerns and is beyond the scope of Lead-DBS, and more studies are required. 

Several limitations of this study must be acknowledged. First, our patient numbers were relatively small, and whether Surgiplan can be considered as ground truth is open to debate, though it is frequently used in DBS surgery. Future studies involving more patients and other DBS electrode localization methods are needed to validate our results. Second, we only used CT and MRI scheduled within days after surgery. Although our new surgical technique (dural puncture) can reduce brain shift, it may still cause error in DBS reconstruction. We used the postoperative image acquired within days after surgery because it is important to reconstruct the electrode soon after surgery to check whether it deviates from or misses the target. This early DBS reconstruction result is of equal significance because it can provide information required by the neurosurgeon to make a timely clinical decision, as discussed above. Nevertheless, future works could focus on postoperative imaging scheduled a few months after the surgery to validate our results and provide more evidence on the accuracy of Lead-DBS. Meanwhile, when calculating the relative distance between the electrode and the STN, the identification of STN may be influenced by the subjectivity of the neurosurgeons, which may cause bias. This result should be validated in future studies, perhaps by segmenting the STN using an automatic algorithm. This may be of great significance with invisible targets such as the globus pallidus internus. Additionally, our optimal contact selection and stimulation parameters are based on the initial follow-up, and we normally program the initial DBS parameter into a monopolar configuration with a relatively low and safe voltage, then as PD progresses, patients may require a higher voltage or bipolar configuration. Our study cannot reflect the value of Lead-DBS-based programming during disease progression, which may explain the lack of a significant difference between patients with both active contacts in the dorsolateral region of the STN and patients with at least one active contact outside the dorsolateral region of the STN. There might be a difference in MDS-UPDRS improvement or change in optimal contact during follow-up, and a long-term study is required. Furthermore, it generally takes several months for patients with DT to benefit from DBS, and future studies are required to evaluate whether the optimal contacts in such patients still overlap with the motor region of the target nucleus in Lead-DBS. 

In conclusion, we found that DBS electrode reconstruction results differed significantly between Lead-DBS and Surgiplan with different postoperative imaging modalities. Compared with Surgiplan, Lead-DBS tends to reconstruct electrode coordinates that are less inferior and more lateral and anterior. However, the variance was only around 1 mm, and Lead-DBS was able to capture the relative position between the electrode and the DBS target. Therefore, the influence of the variance in Lead-DBS is reasonably low, and it can be considered as an accurate postoperative DBS reconstruction method.

## Figures and Tables

**Table 1:** Patient baseline characteristics.

	PD Patients	DT Patients
Number of patients	21	5
Age (year, mean ± SD)	58.4 ± 7.4	53.6 ± 13.0
Gender (male/female)	8/13	4/1
Age of disease onset (year, mean ± SD)	51.1 ± 7.4	47.4 ± 15.5
Disease duration (year, mean ± SD)	7.4 ± 3	6.2 ± 5.1
LEDD (mg/day)	1050.3 ± 589.1	-
**Preoperative assessment**		
UPDRS-III med off (mean ± SD)	44.7 ± 18.9	-
UPDRS-III med on (mean ± SD)	20.5 ± 11.3	-
UPDRS-III improvement (%)	55.4 ± 16.7	-
BFMDRS (mean ± SD)	-	22.7 ± 15.1
GRS (mean ± SD)	-	23 ± 6.7

PD, Parkinson’s disease; DT, dystonia; LEDD, Levodopa equivalent dose per day; UPDRS, Unified Parkinson’s Disease Rating Scale; BFMDRS, Burke–Fahn–Marsden Dystonia Rating Scale; GRS, Global Rating Scale; SD, standard deviation.

**Table 2:** Coordinates calculated using Lead-DBS and Surgiplan with postoperative CT.

		Lead-DBS	Surgiplan	Mean Discrepancy	*p* Value
X coordinate	All	12.36 ± 0.98	12.23 ± 1.11	−0.13	**0.0251**
	Right	12.62 ± 0.91	12.64 ± 1.12	0.02	0.8019
	Left	−12.11 ± 1.00	−11.82 ± 0.95	−0.29	**0.0002**
Y coordinate	All	11.19 ± 1.00	10.03 ± 1.29	−1.16	**<0.0001**
	Right	11.38 ± 1.03	9.86 ± 1.35	−1.52	**<0.0001**
	Left	−11.00 ± 0.94	−10.19 ± 1.24	−0.81	**<0.0001**
Z coordinate	All	2.05 ± 1.09	2.64 ± 1.08	0.59	**<0.0001**
	Right	2.18 ± 1.12	2.65 ± 1.106	0.47	**<0.0001**
	Left	−1.93 ± 1.05	−2.64 ± 1.06	0.71	**0.0012**

## Data Availability

Data are available upon request to the corresponding author after the approval of the local IRB.

## Supplementary Materials

The following supporting information can be downloaded at: https://www.mdpi.com/article/10.3390/jcm12051781/s1, Figure S1: Comparison of calculated electrode coordinates between Lead-DBS and Surgiplan with postoperative CT according to separate hemispheres of the brain; Figure S2: Comparison of the coordinates between Lead-DBS, Surgiplan with postoperative CT, and Surgiplan with MRI. A paired Tukey’s multiple comparisons test found that the X coordi-nates only differed significantly between Surgiplan with postoperative CT and Surgiplan with postoperative MRI (Lead-DBS vs. Surgiplan with postoperative CT, *p* = 0.3367; Lead-DBS vs. Surgiplan with postoperative MRI, *p* = 0.4322; Surgiplan with postoperative CT vs. Surgiplan with postoperative MRI, *p* = 0.038; all paired Tukey’s multiple comparisons test) (A). For the Y coordinates, significant differences were found between Lead-DBS and Surgiplan with postoper-ative CT or MRI (Lead-DBS vs. Surgiplan with postoperative CT, *p* < 0.0001; Lead-DBS vs. Surgiplan with postoperative MRI, *p* < 0.0001; Surgiplan with postoperative CT vs. Surgiplan with postoperative MRI, *p* = 0.9994; all paired Tukey’s multiple comparisons test) (B). For the Z coordinates, significant differences were found between Lead-DBS and Surgiplan with postoper-ative CT or Surgiplan with MRI (Lead-DBS vs. Surgiplan with postoperative CT, *p* < 0.0001; Lead-DBS vs Surgiplan with postoperative MRI, *p* < 0.0001; Surgiplan with postoperative CT vs. Surgiplan with postoperative MRI, *p* = 0.4171; all paired Tukey’s multiple comparisons tests) (C). Error bars indicates standard error; Figure S3: The Lead-DBS reconstructions and postoperative CT and preoperative T2-weighted MRI image of each patient; Figure S4: Euclidean distance between Lead-DBS, Surgiplan, and MER. A paired Dunn’s multiple comparison test suggested that Euclidean distances calculated with the different methods did not show significant differences (Lead-DBS distance vs. Surgiplan distance, *p* > 0.9; Lead-DBS distance vs. MER distance, *p* > 0.9; Surgiplan distance vs. MER distance, *p* > 0.9). Error bars indicate standard error; Figure S5: Clinical improvement following DBS programming and the overlap of the optimal contact with the STN; Figure S6: Clinical improvements in the patients with both active contacts in the dorsolateral region of the STN and patients with at least one active contact outside the dorsolateral region of the STN. The postoperative clinical improvements in the patients with both active contacts in the dorsolateral region of the STN and patients with at least one active contact outside the dorsolateral region of the STN were similar in the on-medication and off-medication states (postoperative off-medication, MDS-UPDRS-III: 21.8 ± 10.7 vs. 21.3 ± 11.1, *p* = 0.98; postoperative on-medication, MDS-UPDRS-III: 10.4 ± 4.63 vs. 10.5 ± 5.2, *p* = 0.77). Table S1: Coordinates calculated using Lead-DBS, Surgiplan with postoperative CT, and Surgiplan with postoperative MRI. Table S2: Euclidean distance between the electrode tip and the pre-defined target.
