# Supplementary material for: Deep Brain Stimulation Electrode Reconstruction: Comparison between Lead-DBS and Surgical Planning System
Source: J Clin Med. 2023 Feb 23;12(5):1781. doi: 10.3390/jcm12051781 (PMC10002993; doi:10.3390/jcm12051781)
Supplement: Supplementary file 1 [file jcm-12-01781-s001.zip › jcm-2185838-supplementary.pdf]

Journal of
Clinical Medicine

MDPI

Supplementary Materials

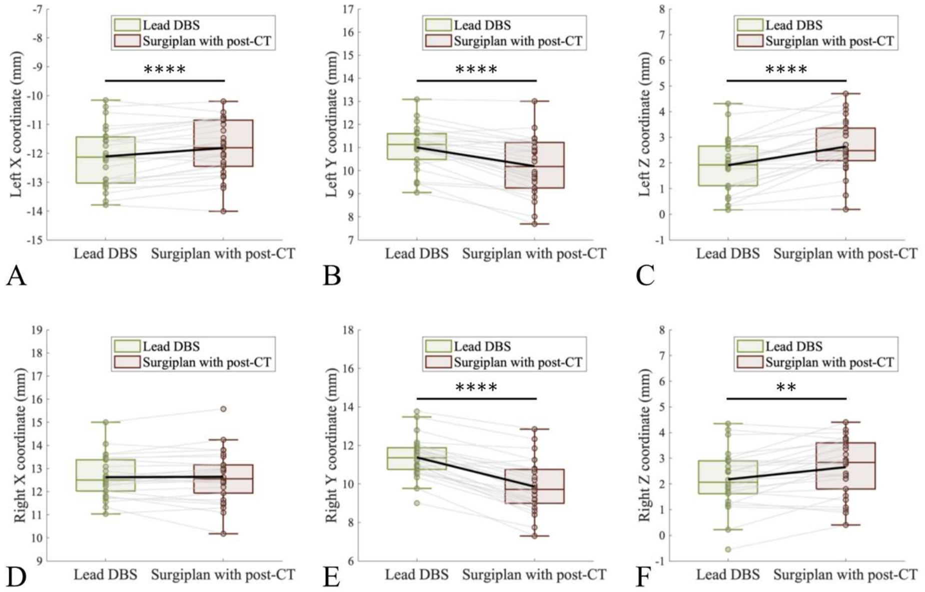

Figure S1. Comparison of calculated electrode coordinates between Lead-DBS and Surgiplan with postoperative CT according to separate hemispheres of the brain. A–C: Coordinates of the left electrode calculated using Lead-DBS and Surgiplan. Significant differences were found for the X, Y, and Z coordinates, with the previously detected trends in differences between the methods persisting (paired *t*-test). D–F: Coordinates of the right electrode calculated using Lead-DBS and Surgiplan. Significant differences were found for the Y and Z coordinates, with the previously detected trends in differences between the methods persisting (paired *t*-test). *: $P<0.05$, **: $P<0.01$, ***: $P<0.001$, ****: $P<0.0001$.

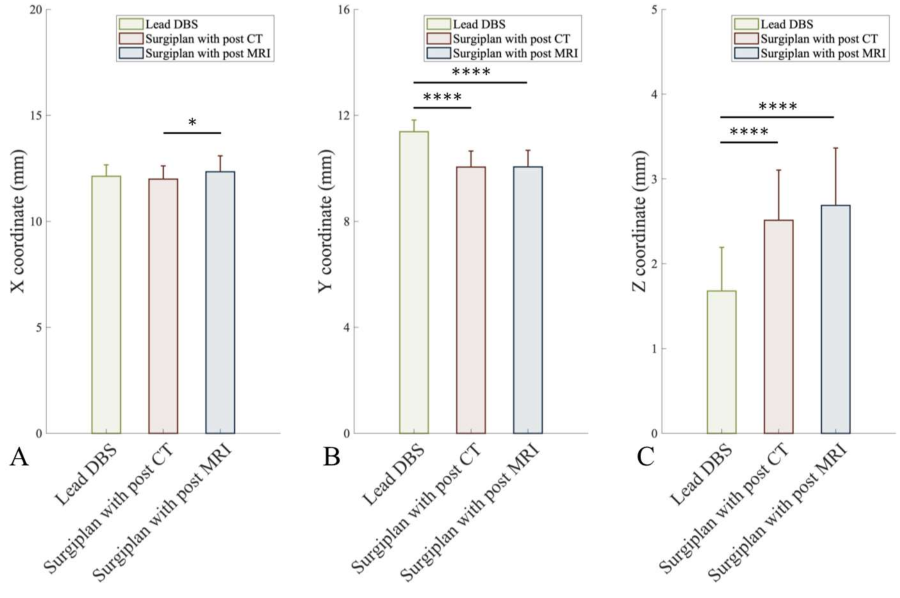

Figure S2. Comparison of the coordinates between Lead-DBS, Surgiplan with postoperative CT, and Surgiplan with MRI. A paired Tukey's multiple comparisons test found that the X coordi-nates only differed significantly between Surgiplan with postoperative CT and Surgiplan with postoperative

MRI (Lead-DBS vs. Surgiplan with postoperative CT, P = 0.3367; Lead-DBS vs. Surgiplan with post-operative MRI, P = 0.4322; Surgiplan with postoperative CT vs. Surgiplan with postoperative MRI, P = 0.038; all paired Tukey's multiple comparisons test) (A). For the Y coordinates, significant differences were found between Lead-DBS and Surgiplan with postoper-ative CT or MRI (Lead-DBS vs. Surgiplan with postoperative CT, P < 0.0001; Lead-DBS vs. Surgiplan with postoperative MRI, P < 0.0001; Surgiplan with postoperative CT vs. Surgiplan with postoperative MRI, P = 0.9994; all paired Tukey's multiple comparisons test) (B). For the Z coordinates, significant differences were found between Lead-DBS and Surgiplan with postoper-ative CT or Surgiplan with MRI (Lead-DBS vs. Surgiplan with postoperative CT, P < 0.0001; Lead-DBS vs Surgiplan with postoperative MRI, P < 0.0001; Surgiplan with postoperative CT vs. Surgiplan with postoperative MRI, P = 0.4171; all paired Tukey's multiple comparisons tests) (C). Error bars indicates standard error. *: P<0.05, **: P<0.01, ***: P<0.001, ****: P<0.0001.

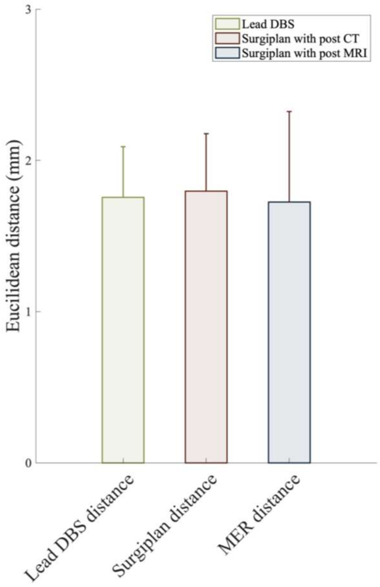

Figure S4. Euclidean distance between Lead-DBS, Surgiplan, and MER. A paired Dunn's multiple comparison test suggested that Euclidean distances calculated with the different methods did not show significant differences (Lead-DBS distance vs. Surgiplan distance, *P* > 0.9; Lead-DBS distance vs. MER distance, *P* > 0.9; Surgiplan distance vs. MER distance, *P* > 0.9). Error bars indicate standard error.

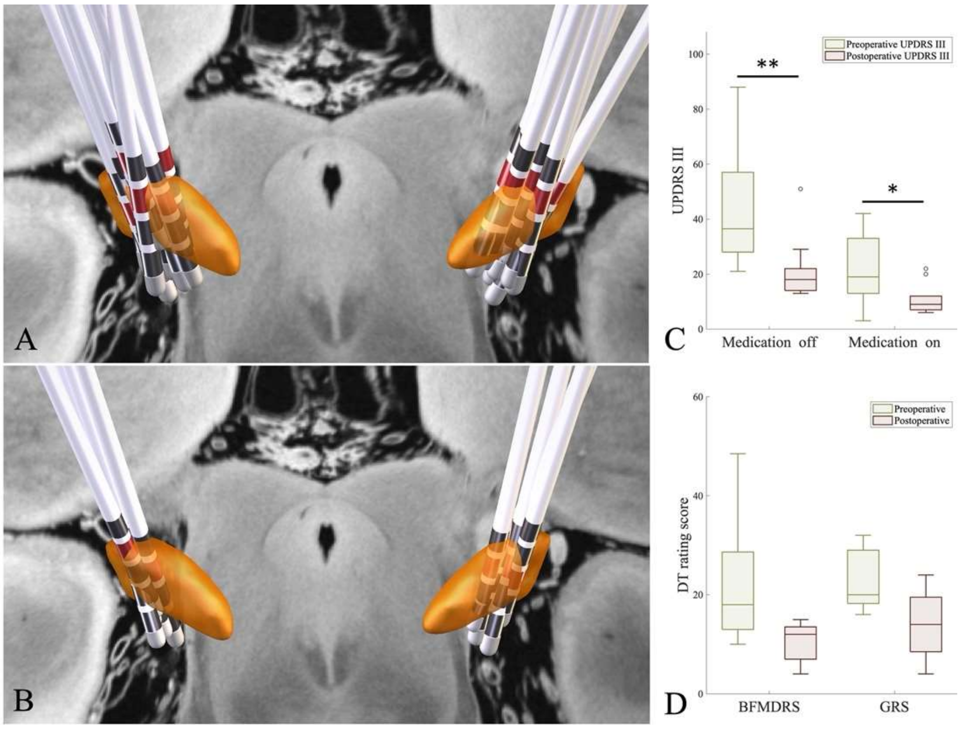

Figure S5. Clinical improvement following DBS programming and the overlap of the optimal contact with the STN. The optimal contact stimulation yielded a significant improvement in PD motor symptoms irrespective of the medication state (two-sample *t*-test, preoperative med-off vs. postoperative med-off, 43.7 ± 19.3 vs. 21.6 ± 11.4, $P = 0.002$; preoperative med-on vs. postoperative med-on: 21.2 ± 12.5 vs. 10.9 ± 5.6, $P = 0.0135$) (C). It also alleviated the motor symptoms in DT patients, although the improvement did not reach the level of significance (preoperative BMFDRS vs. postoperative BMFDRS: 22.5 ± 15.2 vs. 10.4 ± 4.4, $P = 0.116$) (D). After mapping the optimal contacts into the Lead-DBS reconstruction result, all optimal contacts were located within the STN (A and B). The optimal contacts are highlighted in red. *: P<0.05, **: P<0.01, ***: P<0.001, ****: P<0.0001.

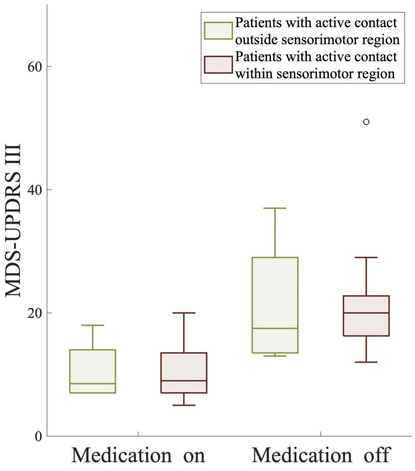

Figure S6. Clinical improvements in the patients with both active contacts in the dorsolateral region of the STN and patients with at least one active contact outside the dorsolateral region of the STN. The postoperative clinical improvements in the patients with both active contacts in the dorsolateral region of the STN and patients with at least one active contact outside the dorsolateral region of the STN were similar in the medication-on and medication-off states (postoperative medication-off, MDS-UPDRS-III: 21.8 ± 10.7 vs. 21.3 ± 11.1, $P = 0.98$; postoperative medication-on, MDS-UPDRS-III: 10.4 ± 4.63 vs. 10.5 ± 5.2, $P = 0.77$).

Supplementary Table S1. Coordinates calculated using Lead-DBS, Surgiplan with postoperative CT, and Surgiplan with postoperative MRI.

	Lead-DBS	Surgiplan with post CT	Surgiplan with post MRI	*P* value		
				Lead-DBS vs Surgiplan with post CT	Lead-DBS vs Surgiplan with post MRI	Surgiplan with post CT vs Surgiplan with post MRI
X coordinate	12.12±1.09	11.99±1.25	12.34±1.51	0.3367	0.4322	**0.038**
Y coordinate	11.38±0.89	10.05±1.21	10.05±1.26	**<0.0001**	**<0.0001**	0.9994
Z coordinate	1.68±1.03	2.51±1.19	2.69±1.35	**<0.0001**	**0.0003**	0.4171

* The coordinate information was obtained from only the subset of patients with postoperative MRI, postoperative CT, and Lead-DBS data available.

Supplementary Table S2. Euclidean distance between the electrode tip and the pre-defined target.

	Lead-DBS (min, max)	Surgiplan (min, max)	MER-recorded distance (min, max)	*P* value		
				Lead-DBS vs Surgiplan with post CT	Lead-DBS vs Surgiplan with post MRI	Surgiplan with post CT vs Surgiplan with post MRI
Euclidean distance	1.75±0.67 (0.36, 3.17)	1.80±0.76 (0.51, 4)	1.72±1.20 (0, 4.5)	>0.9999	>0.9999	>0.9999
